# Cholesterol Efflux Capacity Associates with the Ankle-Brachial Index but Not All-Cause Mortality in Patients with Peripheral Artery Disease

**DOI:** 10.3390/diagnostics11081407

**Published:** 2021-08-04

**Authors:** Robert K. Clemens, Monika Hunjadi, Andreas Ritsch, Lucia Rohrer, Thomas O. Meier, Beatrice Amann-Vesti, Arnold von Eckardstein, Wijtske Annema

**Affiliations:** 1 Vascular Center, Cantonal Hospital Baden, University of Zurich, Im Ergel 1, CH-5404 Baden, Switzerland; 2 Department of Internal Medicine, Medical University of Innsbruck, 6020 Innsbruck, Austria; monika.hunjadi@i-med.ac.at (M.H.); andreas.ritsch@i-med.ac.at (A.R.); 3 Institute of Clinical Chemistry, University Hospital Zurich, University of Zurich, Rämistrasse 100, CH-8091 Zurich, Switzerland; lucia.rohrer@usz.ch (L.R.); arnold.voneckardstein@usz.ch (A.v.E.); wijtske.wallimann@usz.ch (W.A.); 4 Clinic for Angiology, University Hospital Zurich, University of Zurich, Rämistrasse 100, CH-8091 Zurich, Switzerland; thomas.meier@usz.ch; 5 Angiology, Clinic in the Park, Seestrasse 220, CH-8027 Zurich, Switzerland; beatrice.amann@hirslanden.ch

**Keywords:** cholesterol efflux capacity, high-density lipoprotein, peripheral artery disease, risk prediction

## Abstract

Background: Cholesterol efflux is an important mechanism by which high-density lipoproteins (HDLs) protect against cardiovascular disease. As peripheral artery disease (PAD) is associated with high mortality rates, mainly due to cardiovascular causes, we investigated whether cholesterol efflux capacity (CEC) of apolipoprotein B (apoB)-depleted plasma, a widely used surrogate of HDL function, may serve as a predictive marker for mortality in this patient population. Methods: In this prospective single-center study (median follow-up time: 9.3 years), apoB-containing lipoproteins were precipitated from plasma of 95 patients with PAD and incubated with J744-macrophages, which were loaded with radiolabeled cholesterol. CEC was defined as the fractional radiolabel released during 4 h of incubation. Results: Baseline CEC was lower in PAD patients that currently smoked (*p* = 0.015) and had a history of myocardial infarction (*p* = 0.011). Moreover, CEC showed a significant correlation with HDL-cholesterol (*p* = 0.003) and apolipoprotein A-I levels (*p* = 0.001) as well as the ankle-brachial index (ABI, *p* = 0.018). However, CEC did not differ between survivors and non-survivors. Neither revealed Kaplan–Meier and Cox regression analyses any significant association of CEC with all-cause mortality rates. Conclusion: Taken together, CEC is associated with ABI but does not predict all-cause mortality in patients with PAD.

## 1. Introduction

Peripheral artery disease (PAD) is a very prevalent condition in developed countries. Around one in five people over the age of 65 suffer from PAD [1] and the number of PAD patients is expected to increase facing the growing elderly population. Individuals with PAD are at high risk for morbidity and mortality, especially caused by cardiovascular events like myocardial infarction, stroke, and critical limb ischemia [2]. According to data from the Reduction of Atherothrombosis for Continued Health (REACH) registry patients with PAD exhibited a cardiovascular event rate (composite of myocardial infarction, stroke, vascular death, and rehospitalization due to vascular events) of 21% after one year of follow-up and increasing to 40% at 3 years [3]. Notably, the probability of patients with PAD experiencing a future cardiovascular event was even higher as patients with coronary artery disease or cerebrovascular disease [3]. Accurate and early identification of PAD patients at high risk of future cardiovascular morbidity and mortality is therefore essential. However, the high comorbidity rates and the many therapeutic interventions among this group of patients make outcome prediction with conventional risk factors, such as smoking, diabetes, hypertension, and hyperlipidemia, challenging. Consequently, alternative or additional markers are required to improve risk stratification.

The ability of high-density lipoprotein (HDL) particles to remove cholesterol from lipid-laden macrophages, the first step in reverse cholesterol transport, is a crucial mechanism by which HDLs protect against atherosclerotic cardiovascular disease (CVD) [4,5]. Recent findings highlight the importance of HDL functionality measurements, particularly in cholesterol efflux capacity (CEC), over measurement of HDL-cholesterol (HDL-C) levels for cardiovascular risk assessment. Studies based on samples from the general population found that CEC is inversely associated with future cardiovascular events, independent of HDL-C concentration and other traditional cardiovascular risk factors [6,7,8,9]. Likewise, the ability of apolipoprotein B (apoB)-depleted serum to accept cholesterol from macrophages was a predictor of mortality in patients with established coronary artery disease [10,11,12,13]. It has not yet been investigated whether macrophage-specific CEC is linked to outcome in patients with PAD. Therefore, the aim of this study was to assess whether HDL CEC could predict mortality in PAD patients and to explore which factors impact CEC in this specific patient population.

## 2. Materials and Methods

This single-center prospective cohort study included 95 patients with PAD (73 male and 22 female) with or without a history of peripheral vascular intervention or vascular surgery. PAD was confirmed either angiographically, sonographically, and/or with ankle-brachial index (ABI)-measurement. Patients were recruited between 2002 and 2005 and were followed up until 2016. The mean age at enrollment was 68.3 years (range 47.3–86.3 years). A primary visit was performed at which written informed consent was obtained from all patients after proper explanation of the study and its purpose to the patients. In the case where patients were willing to participate, they were invited for a second study visit. All subjects could withdraw from the study at any time. The study protocol was approved by the Cantonal Ethics Committee of Zurich and is in line with the principles of the Declaration of Helsinki.

Non-Laboratory Measurements and Definitions: Information concerning the medical history, current medication, previous cardiovascular and cerebrovascular events, hypertension, dyslipidemia, and diabetes mellitus were obtained from all subjects at the initial screening visit by interviewing the patient and reviewing the electronic hospital chart. Diabetes was defined as fasting serum glucose ≥7 mmol/L or use of oral antidiabetics or insulin. Hyperlipidemia was defined as use of lipid-lowering drugs or total cholesterol >5.2 mmol/L and/or triglycerides >2.26 mmol/L. Hypertension was defined as a systolic blood pressure ≥140 mm Hg and/or a diastolic blood pressure ≥90 mm Hg. Smoking was defined as active cigarette smoking. The body mass index was calculated as weight in kilograms divided by height in meters squared. The ABI and carotid intima-media thickness (cIMT) were measured in all participants at the time of the second visit. The ABI was determined with the patient in supine position after 20 min of rest using the Vicorder system (Skidmore Medical Ltd., Bristol, UK). The systolic pressure was obtained in both arms and at the tibial anterior as well as tibial posterior artery in each ankle. The ABI was calculated by dividing the highest ankle pressure by the highest systolic pressure of the arm. The cIMT was assessed using a high-resolution ultrasound transducer and semiautomatic edge-detection software (GE Healthcare, Glattbrugg, Switzerland). The cIMT of the dorsal wall of both common carotid arteries, proximal to the carotid bulb, was measured according to international recommendations [14].

The Fontaine-stage classification was used to grade the severity of the clinical symptoms: stage I asymptomatic, stage IIa claudication at distance >200 m, and stage IIb claudication at distance <200 m. Three patients had a complicated PAD with minor ulcer but no resting pain (stage IIc).

Laboratory Measurements: All laboratory measurements were performed using the baseline lithium-heparin plasma samples available from the second visit between 2002 and 2004. Total cholesterol was measured using the cholesterol oxidase-4 aminophenazone (CHOD-PAP) method, HDL-cholesterol using the cholesterol esterase/cholesterol oxidase/4-aminoantipyrine method, triglycerides using the glycerine phosphate oxidase-4-aminophenazone (GPO-PAP) method, C-reactive protein (CRP) using an immunoturbimetric assay, and creatinine using a modified version of the Jaffé method, respectively, all on a cobas 8000 modular analyzer (Roche Diagnostics, Rotkreuz, Switzerland). LDL-cholesterol was calculated with the Friedewald formula [15]. Non-HDL-cholesterol was derived by subtracting HDL-cholesterol from total cholesterol. The chronic kidney disease epidemiology collaboration (CKD-EPI, 2009) equation was used to estimate the glomerular filtration rate [16]. Plasma apolipoprotein B (apoB) and apoA-I were determined by immunonephelometric assays on a BN Prospec System (Siemens Healthcare Diagnostics, Erlangen, Germany). Lipoprotein(a) (Lp(a)) was measured with an immunoturbidimetric assay (Randox, Crumlin, UK) on a Konelab 30 clinical chemistry analyzer (Thermo Fisher Diagnostics, Pratteln, Switzerland). Serum homocysteine was determined using a chemiluminescence microparticle assay on an Architect i2000SR immunoassay analyzer (Abbott, Baar, Switzerland).

Assessment of Cholesterol Efflux Capacity: To obtain the apoB-free fraction, apoB-containing lipoproteins were precipitated from K-EDTA plasma obtained at the initial visit and biobanked at −80 °C until analysis using a mix of 8.2% tungstophosphoric acid hydrate (Merck, Darmstadt, Germany) and 6.2% 1 M MgCl_2_ (Roth, Karlsruhe, Germany). CEC was quantitated as reported previously [13,17,18]. The murine macrophage cell line J774.1A (ATCC, Rockville, MD, USA) was cultured in Dulbecco’s Modified Eagle Medium (Sigma-Aldrich, St. Louis, MO, USA) supplemented with 1% fetal bovine serum at 37 °C in a humidified incubator (5% CO_2_, 95% air). For efflux experiments, cells were seeded in 96-well plates (50,000 cells per well). The J744 macrophages were then loaded overnight with 2.5 µCi/mL ^3^H-cholesterol followed by equilibration in serum-free growth medium containing 0.2% serum albumin. To induce ATP-binding cassette transporter A1 (ABCA1) expression J774 cells were stimulated with 0.3 mM N6,2′-0-dibutyryladenosine 3′,5′-cyclic monophosphate (cAMP, Sigma Aldrich, St. Louis, MO, USA). Thereafter, efflux medium containing 2.8% apoB-depleted plasma was added to the macrophages for 4 h. All steps were performed in the presence of 5 µg/mL acyl-coenzyme A cholesterol acyltransferase inhibitor (Santa Cruz Biotechnology, Santa Cruz, CA, USA).

The effluxed cholesterol label was quantified by liquid scintillation counting. Values obtained from control cells without added apoB-depleted plasma were subtracted to correct for passive diffusion. Efflux per well was calculated according the following formula: [(counts released into the medium containing 2.8% apoB-free plasma—counts released into the medium without acceptor)/counts recovered from the cells extracted before the efflux experiment] × 100. Values were normalized to values obtained with a pooled plasma control run with each plate to correct for interassay variation across plates. Each sample was run in triplicate.

Statistical Analyses: Statistical analyses were performed using IBM SPSS Statistics version 26.0 (IBM Corp, Armonk, NY, USA) and GraphPad Prism version 8.0.0 (GraphPad Software Inc., San Diego, CA, USA). Normally distributed continuous variables are presented as mean ± standard deviation, whereas continuous variables with a skewed distribution are summarized as medians with 25th and 75th percentiles. Categorical variables are given as numbers (percentages). Patient characteristics were analyzed separately for gender-stratified tertiles of CEC. Differences between groups were tested for statistical significance with one-way analysis of variance with subsequent Bonferroni post-hoc testing for multiple comparisons for normally distributed continuous variables, with the Kruskal–Wallis test for variables with a skewed distribution, and the chi-square test for categorical variables. Gender-adjusted linear regression analysis was carried out to determine the contribution of variables to CEC. Log-transformation was used for variables with a skewed distribution. Kaplan–Meier survival curves were plotted to evaluate the association of CEC with mortality. The log-rank test was used to assess statistical differences among the survival curves of the different cholesterol efflux tertiles. Univariate and multivariate Cox proportional hazard regression analyses were employed to test the influence of CEC on mortality. The results are reported as hazard ratios (HRs) per 1-standard deviation increase with corresponding 95% confidence intervals (95% CIs). Statistical significance was defined as *p* < 0.05.

## 3. Results

### 3.1. Patient Characteristics

In this single-center prospective cohort study, 95 PAD patients were enrolled between 2002 and 2004. At follow-up, in 2016, 50 of these patients were alive, 31 of these patients were dead, and 13 patients were lost to follow-up. Patients that died during follow-up were older and had a higher prevalence of diabetes mellitus. The sample volume was insufficient for the cholesterol efflux measurement in one patient. Therefore, 81 patients were included in the final data analysis. Participants were divided into gender-stratified tertiles according to CEC. The median cholesterol efflux value was 85.0 (interquartile range (IQR) 79.0–88.5) in the first tertile, 99.0 (IQR 95.5–102.0) in the second tertile, and 118.0 (IQR 111.0–135.0) in the third tertile (Table 1). PAD patients in the lowest tertile of CEC had significantly lower HDL-C- and apoA-I-levels as compared to PAD patients in the highest tertile of CEC (1.16 ± 0.36 mmol/L vs. 1.42 ± 0.41 mmol/L for HDL-C, *p* = 0.030, and 1.36 ± 0.24 g/L vs. 1.56 ± 0.28 g/L for apoA-I, *p* = 0.009, Table 1). Moreover, these patients had lower ABI values (0.76 ± 0.17 vs. 0.89 ± 0.18, *p* = 0.039, Table 1). Patients in the lowest tertile had significantly more often a history of previous myocardial infarction (MI) compared to patients in the mid and highest tertile (44% vs. 19%, *p* = 0.017, and 44% vs. 15%, *p* = 0.040, Table 1). cIMT did not differ between tertiles of CEC (Table 1).

### 3.2. Determinants of CEC in PAD Patients

We next sought to define factors that influence CEC in patients with PAD. In all study participants, CEC tended to be lower in males than in females (98.5 (87.0–110.0) vs. 105.0 (96.0–119.0), *p* = 0.051, Supplemental Figure S1a). Additionally, CEC was significantly lower in current smokers than in non-smokers (95.0 (85.0–108.0) vs. 102.5 (94.0–117.5), *p* = 0.015, Supplemental Figure S1b) and in patients with a history of MI than in patients without a history of MI (89.0 (85.0–101.0) vs. 102.0 (92.5–117.0), *p* = 0.011, Supplemental Figure S1c). However, there was no significant difference in CEC between PAD patients with or without a history of a cerebrovascular accident (CVA, *p* = 0.132, Supplemental Figure S1d), with or without diabetes (*p* = 0.722, Supplemental Figure S1e), with or without dyslipidemia (*p* = 0.721, Supplemental Figure S1f), or with or without hypertension (*p* = 0.817, Supplemental Figure S1g). Because of the gender differences in CEC, gender-adjusted regression analysis was used to determine the relationships of CEC with clinical and biochemical variables. In agreement with the analyses of patient characteristics categorized by tertiles of cholesterol efflux, CEC in PAD patients showed a strong correlation with plasma HDL-C (β = 0.333, *p* = 0.003, Table 2) and apoA-I levels (β = 0.363, *p* = 0.001, Table 2). Moreover, CEC correlated positively with ABI (β = 0.258, *p* = 0.018, Table 2) and this association remained significant after further adjustment for HDL-C (β = 0.247, *p* = 0.017) or apoA-I levels (β = 0.204, *p* = 0.049). On the other hand, CEC was not related to age, BMI, other lipid parameters, plasma CRP, eGFR, and cIMT (Table 2).

### 3.3. Association of Cholesterol Efflux with All-Cause Mortality

**Figure 1:**
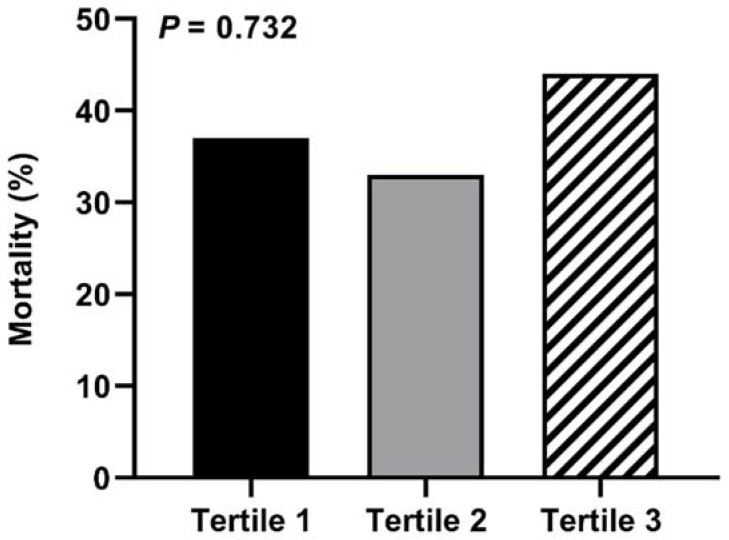
Mortality according to gender-stratified tertiles of CEC.

**Figure 2:**
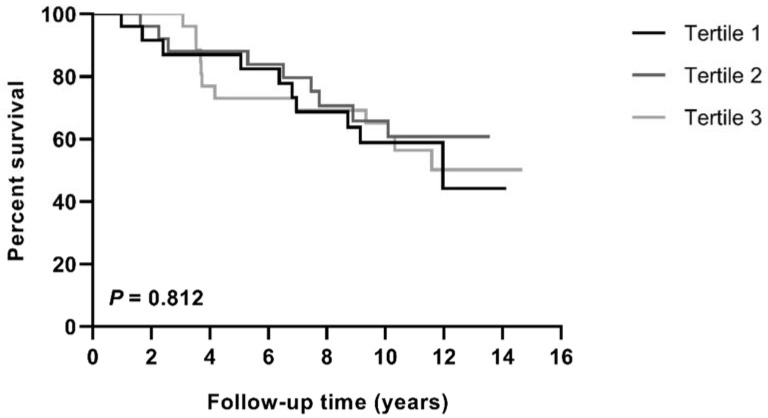
Kaplan–Meier curves for mortality by gender-stratified tertiles of CEC. Statistical difference was determined using the log-rank test.

Median follow-up time was 9.3 years (range 0.4–14.7 years). At the end of the follow-up period, 10 patients (37%) died in the first tertile of CEC, 9 patients (33%) in the second tertile, and 12 patients in the third tertile (44%, *p* = 0.732, Figure 1). Similarly, Kaplan–Meier survival curves revealed no difference in all-cause mortality rates over time among the different tertiles of CEC (Figure 2, *p* = 0.812). As shown in Table 3, the unadjusted HR (95% CI) for all-cause mortality per 1-SD increase in CEC was 0.843 (0.587–1.208) (*p* = 0.352). Adding gender to the model did not significantly change the calculated HR (0.867 (0.599–1.255), *p* = 0.449, Table 3). CEC remained unrelated to all-cause mortality after adjustment for other potential confounders (Table 3). Of note, also none of the classic lipid parameters, i.e., cholesterol, LDL-C, HDL-C, non-HDL-C, triglycerides, apoA-I, apoB, and Lp(a), predicted mortality in our cohort of patients with PAD (Supplemental Table S1–S8).

## 4. Discussion

PAD is associated with an elevated risk of cardiovascular events and shortened life expectancy [19]. PAD patients regularly have polyvascular disease and exhibit a cardiovascular event rate of 40% within three years after first diagnosis [1]. Since conventional risk factors inadequately identify PAD patients at high risk of poor prognosis, better risk prediction tools based on novel biomarkers are urgently needed. The development of targeted therapies may help reduce cardiovascular events and hence, premature mortality in PAD patients. In this study, we investigated the predictive value of CEC, an important antiatherosclerotic functionality of HDLs, for mortality in PAD and determining factors influencing HDL cholesterol efflux potential in this patient population.

Currently, recommendations for therapies in PAD patients are based on a risk assessment that measures the severity of PAD with traditional markers such as ABI. Other indicators such as the cIMT are under discussion. While patients with a low ABI (<0.4) have a high risk for cardiovascular events and mortality, it is more uncertain in patients suffering from an intermediately impaired arterial perfusion of the lower legs (ABI between 0.4–0.9). We previously found that traditional markers of peripheral atherosclerosis, ABI and cIMT, did not predict mortality in the PAD patient population used in this study [20]. This is in concordance with other recent studies that questioned the predictive value of cIMT in PAD patients [21,22]. There are new promising biomarkers, like the cardiac markers heart-type fatty acid binding protein, highly sensitive troponin T, and N-terminal pro b-type natriuretic peptide, and the inflammatory marker C-reactive protein, that have received considerable attention for the prognosis of PAD patients in recent years [20,23,24,25]. However, these biomarkers are not yet used in clinical routine for cardiovascular risk assessment in PAD patients.

A reduced HDL functionality, particularly in CEC, is increasingly recognized as an important cardiovascular risk factor. The efflux of excessive cholesterol from arterial macrophage foam cells towards HDLs is the first step in the reverse cholesterol transport pathway, facilitating the removal of cholesterol from the body and key in the protection of HDLs against atherosclerotic cardiovascular disease [4,5]. Previous studies reported that a lower CEC is associated with an increased incidence of cardiovascular events and mortality in the general population as well as patients with established coronary artery disease [6,7,8,10,11,12,13]. Furthermore, two comprehensive meta-analyses indicated that individuals with a higher CEC have a reduced risk for major adverse cardiovascular events and all-cause mortality [9,26]. Contrary to these observations, we did not find any association between CEC and all-cause mortality in PAD patients in the current study, even after adjustment for other potential confounders. Notably, an inverse association between CEC and adverse outcome is not present in all published studies. Our findings agree with a recent study indicating that CEC had no predictive value for all-cause and cardiovascular mortality in renal transplant recipients, another patient group with high cardiovascular mortality rates and failure of traditional risk factors to adequately predict events [27]. Cahill et al. [28] measured CEC in a nested case-control study of the Health Professionals Follow-up Study (HPFS) and found no independent relationship between CEC and coronary heart disease events. Furthermore, in a cohort of 1150 patients with suspected stable coronary artery disease undergoing elective angiography, an increased CEC was paradoxically associated with a higher risk for incident major adverse cardiovascular events, myocardial infarction, and stroke during a follow-up of 3 years [29]. It is important to note that the comparability of published clinical studies is limited due to the different assay protocols used to measure CEC and these methodological differences may contribute to these discrepant results.

We have examined for the first time in a prospective cohort study the association of CEC with mortality in PAD patients. Previous research in 1255 participants of the Multi-Ethnic Study of Atherosclerosis reported that cholesterol efflux to apoB-depleted serum did not differ between patients with or without PAD [30]. The same authors showed that CEC was not associated with the development of clinical PAD and could not predict a future decline in ABI [30]. Another study suggested that the relationship between CEC and PAD is influenced by diabetes status since an impairment in CEC in the presence of PAD was only observed in patients without diabetes or newly diagnosed diabetes but not in patients with established diabetes [31]. Recently, investigators have examined the effects of exercise training in PAD patients and showed that neither 24 weeks of supervised treadmill exercise nor lower extremity resistance training improved HDL CEC in individuals with PAD [32].

Another important finding that emerged from the present study is that CEC correlated with ABI and this relationship remained robust after correction for HDL-C and apoA-I levels. ABI is not only a marker of lower limb PAD but may also reflect the degree of systemic atherosclerosis. These data are consistent with the concept that cholesterol efflux from macrophages decreases foam cell formation and slows atherosclerosis development. Therefore, a reduced potential to remove excess cholesterol from macrophage foam cells may lead to a higher coronary and peripheral atherosclerotic burden in PAD patients. Interestingly, the lack of a significant association between CEC and cIMT, another surrogate indicator of subclinical atherosclerosis, does not support this hypothesis. A possible explanation for this might be that the cIMT increases continuously with age and is linked to other cardiovascular risk factors, like BMI, blood pressure, total cholesterol, triglycerides, glucose, and smoking [33]. Additionally, it has been suggested that carotid plaque burden is a better predictor of cardiovascular risk than cIMT [21,22,34].

Although our current study focused on CEC, it is essential to compare our observations with the impact of classic lipid markers on cardiovascular risk in PAD patients. Dyslipidemia is a risk factor for both the manifestation and progression of PAD [35]. The 2017 European Society of Cardiology guidelines on the treatment of peripheral artery disease were updated in 2020 and state that an LDL-C target of <1.4 (1.8) mmol/L or at least a 50% decrease in baseline LDL-C should be achieved in all PAD patients [19,36]. Substantial evidence from randomized clinical trials shows that statin therapy in patients with PAD reduces the risk of major adverse cardiovascular events by approximately 20% [37,38]. Among the conventional lipid risk factors, the ratio of total to HDL-C emerged as the strongest determinant of large vessel PAD in both the Physician’s Health Study and the San Diego Population Study [39,40,41]. Low levels of HDL-C may predispose to adverse outcomes as a perspective study including 254 patients suffering from PAD concluded that patients with reduced HDL-C levels (<1.0 mmol/L for men and <1.3 mmol/L for women) presented with higher incidences of mortality and major cardiovascular events over a 3-year period [42]. In our study population, none of the traditional lipid parameters was a useful biomarker for the early prediction of mortality in PAD patients.

Our study as several potential limitations. First, the generalizability is limited by the relatively small sample size and the analyses of single-center data. We cannot exclude that our sample size was too small to identify CEC as an independent predictor of all-cause mortality in PAD patients and our results may not be transferable to a multicenter setting. Another issue with the study design is that we investigated all-cause mortality and not mortality due to major cardiovascular events. However, we were not able to perform analyses specifically for cardiovascular mortality, as no data on the cause of death were available in the current study. Additional to these considerations, the studied group of PAD patients suffered from an intermediately impaired arterial perfusion and did not include patients with severer forms of PAD, which are at very high risk for cardiovascular events and death. Although our work provides important insights into the role of CEC in risk prediction in PAD patients, the current findings need to be confirmed in a larger multicenter setting. Future studies should also include patients with severe PAD (ABI < 0.4) as well as patients with non-compressible arteries (ABI > 1.4) and the analyses should be extended to major cardiovascular events and cardiovascular mortality.

## 5. Conclusions

In summary, the present study has demonstrated that CEC was associated with ABI but not with mortality in patients with intermediate PAD. As long as there is uncertainty about the value of biomarkers for predicting cardiovascular mortality in PAD patients, a combination of biomarkers should be considered when stratifying the individual risk of cardiovascular events.

## Figures and Tables

**Table 1:** Patient characteristics by gender-stratified tertiles of CEC.

	First (*n* = 27)	Second (*n* = 27)	Third (*n* = 27)	*p*-Value
CEC	85.0 (79.0–88.5)	99.0 (95.5–102.0)	118.0 (111.0–135.0)	<0.001
**Patient Characteristics**				
Male gender, *n* (%)	22 (82)	21 (78)	21 (79)	0.928
Age, years	68.3 ± 8.9	68.5 ± 11.5	66.2 ± 8.9	0.648
Smoking, *n* (%)	19 (70)	12 (44)	14 (52)	0.142
History of MI, *n* (%)	12 (44)	5 (19) ^a^	4 (15) ^b^	0.026
History of CVA, *n* (%)	15 (56)	12 (44)	11 (41)	0.525
Diabetes, *n* (%)	10 (37)	5 (19)	7 (26)	0.306
Dyslipidemia, *n* (%)	21 (78)	18 (67)	23 (85)	0.271
Hypertension, *n* (%)	22 (82)	21 (78)	22 (82)	0.925
BMI, kg/m^2^	25.6 (24.1–28.5)	25.7 (23.3–29.0)	24.0 (22.0–27.8)	0.223
**Lipid Profile**				
Cholesterol, mmol/L	4.2 ± 1.1	4.5 ± 1.1	4.6 ± 0.9	0.317
HDL-cholesterol, mmol/L	1.16 ± 0.36	1.25 ± 0.34	1.42 ± 0.41 ^b^	0.033
LDL-cholesterol, mmol/L	2.5 ± 1.8	2.7 ± 0.6	3.0 ± 0.8	0.665
Non-HDL cholesterol, mmol/L	3.0 ± 1.2	3.3 ± 1.0	3.2 ± 0.8	0.721
Triglycerides, mmol/L	1.95 (1.51–3.00)	1.50 (0.98–1.99)	2.01 (1.12–2.52)	0.051
ApoA-I, g/L	1.36 ± 0.24	1.48 ± 0.21	1.56 ± 0.28 ^b^	0.012
ApoB, g/L	0.94 ± 0.17	0.90 ± 0.22	0.92 ± 0.19	0.688
Lp(a), mg/L	224 (77–701)	150 (47–212)	146 (38–565)	0.401
Homocysteine, µmol/L	14.7 (12.2–19.3)	13.7 (11.5–16.0)	12.9 (10.7–15.0)	0.171
**Inflammation**				
CRP, mg/L	4.1 (2.2–6.3)	3.1 (1.6–6.4)	2.8 (1.3–4.8)	0.424
**Kidney Function**				
eGFR, ml/min/1.73 m^2^	67 ± 20	70 ± 20	63 ± 21	0.410
**Extent of PAD**				
ABI	0.76 ± 0.17	0.80 ± 0.23	0.89 ± 0.18 ^b^	0.042
cIMT, mm	0.78 ± 0.13	0.77 ± 0.16	0.76 ± 0.11	0.792
Fontaine stage				0.596
Stage I	11 (41)	10 (37)	11 (41)	
Stage IIa	7 (26)	11 (41)	12 (44)	
Stage IIb	8 (30)	4 (15)	4 (15)	
Stage IIc	1 (4)	2 (7)	0 (0)	
Stage III	0 (0)	0 (0)	0 (0)	
Stage IV	0 (0)	0 (0)	0 (0)	

Data are expressed as mean ± standard deviation, median [interquartile range] or total (percentage). Differences between the groups were tested with one-way analysis of variance followed by Bonferroni post hoc test for normally distributed variables, Kruskal–Wallis test for variables with a skewed distribution, and chi-square test for categorical variables. ^a^ *p* < 0.05 first vs. second tertile, ^b^ *p* < 0.05 first vs. third tertile. Abbreviations: MI, myocardial infarction; CVA, cerebrovascular accident; BMI, body mass index; HDL, high-density lipoprotein; LDL, low-density lipoprotein; apoA-I, apolipoprotein A-I; apoB, apolipoprotein B; Lp(a), lipoprotein(a); CRP, C-reactive protein; eGFR, estimated glomerular filtration rate; PAD, peripheral artery disease; ABI, ankle brachial index; IMT, intima-media thickness.

**Table 2:** Relationship of traditional cardiovascular risk factors with CEC in patients with peripheral artery disease.

Determinant	β Coefficient	*p*-Value
**Patient Characteristics**		
Age	−0.149	0.183
BMI	−0.164	0.134
**Lipid Profile**		
Cholesterol	0.028	0.800
HDL-cholesterol	0.333	0.003
Non-HDL cholesterol	−0.093	0.398
LDL-cholesterol	−0.112	0.616
Triglycerides	−0.155	0.158
ApoA-I	0.363	0.001
ApoB	−0.060	0.584
Lp(a)	−0.179	0.119
Homocystein	−0.161	0.154
**Inflammation**		
CRP	−0.141	0.204
**Kidney Function**		
eGFR	−0.186	0.089
**Extent of PAD**		
ABI	0.258	0.018
cIMT	−0.085	0.456

Gender-adjusted standardized regression coefficients (β) are shown. Not normally distributed parameter were log transformed. Abbreviations: MI, myocardial infarction; CVA, cerebrovascular accident; BMI, body mass index; HDL, high-density lipoprotein; LDL, low-density lipoprotein; apoA-I, apolipoprotein A-I; apoB, apolipoprotein B; Lp(a), lipoprotein(a); CRP, C-reactive protein; eGFR, estimated glomerular filtration rate; PAD, peripheral artery disease; ABI, ankle brachial index; IMT, intima-media thickness.

**Table 3:** Hazard ratios for mortality by CEC.

	HR Per 1-SD Increment [95%CI]	*p*-Value
Crude	0.843 (0.587–1.208)	0.352
Corrected for gender	0.867 (0.599–1.255)	0.449
Corrected for age and gender	0.925 (0.639–1.340)	0.680
Corrected for age, gender, and HDL-C	0.953 (0.651–1.398)	0.807
Corrected for age, gender, and ABI	0.932 (0.625–1.389)	0.729
Corrected for age, gender, and smoking	0.992 (0.675–1.457)	0.967

Data are expressed as hazard ratios (HR) per 1 standard deviation (SD) increment and their respective 95% confidence intervals (CIs).

## Data Availability

The data presented in this study are available on request from the corresponding author.

## Supplementary Materials

The following are available online at https://www.mdpi.com/article/10.3390/diagnostics11081407/s1.
