# Supplementary material for: Cholesterol Efflux Capacity Associates with the Ankle-Brachial Index but Not All-Cause Mortality in Patients with Peripheral Artery Disease
Source: Diagnostics (Basel). 2021 Aug 4;11(8):1407. doi: 10.3390/diagnostics11081407 (PMC8394478; doi:10.3390/diagnostics11081407)
Supplement: Supplementary file 1 [file diagnostics-11-01407-s001.zip › diagnostics-1265406-supplementary.pdf]

Supplementary data to Clemens et al., Cholesterol Efflux Capacity Associates with the Ankle-Brachial Index but Not All-Cause Mortality in Patients with Peripheral Artery Disease

Supplemental Table S1: Hazard ratios for mortality by cholesterol.

	**HR Per 1-SD Increment [95%CI]**	***p*-Value**
Crude	0.817 [0.591-1.129]	0.220
Corrected for gender	0.799 [0.564-1.130]	0.204
Corrected for age and gender	0.784 [0.563-1.091]	0.149
Corrected for age, gender, and ABI	0.773 [0.546-1.093]	0.145
Corrected for age, gender, and smoking	0.790 [0.567-1.102]	0.165

Data are expressed as hazard ratios (HR) per 1 standard deviation (SD) increment and their respective 95% confidence intervals (CIs).

Supplemental Table S2: Hazard ratios for mortality by low-density lipoprotein cholesterol.

	**HR Per 1-SD Increment [95%CI]**	***p*-Value**
Crude	0.847 [0.434-1.654]	0.628
Corrected for gender	0.766 [0.353-1.665]	0.766
Corrected for age and gender	0.748 [0.359-1.560]	0.439
Corrected for age, gender, and ABI	0.698 [0.307-1.588]	0.391
Corrected for age, gender, and smoking	0.746 [0.346-1.609]	0.454

Data are expressed as hazard ratios (HR) per 1 standard deviation (SD) increment and their respective 95% confidence intervals (CIs).

Supplemental Table S3: Hazard ratios for mortality by high-density lipoprotein cholesterol.

	**HR Per 1-SD Increment [95%CI]**	***p*-Value**
Crude	0.778 [0.529-1.143]	0.201
Corrected for gender	0.804 [0.541-1.195]	0.280
Corrected for age and gender	0.856 [0.560-1.307]	0.470
Corrected for age, gender, and ABI	0.769 [0.491-1.209]	0.253
Corrected for age, gender, and smoking	0.914 [0.585-1.430]	0.695

Data are expressed as hazard ratios (HR) per 1 standard deviation (SD) increment and their respective 95% confidence intervals (CIs).

Supplemental Table S4: Hazard ratios for mortality by non-high-density lipoprotein cholesterol.

	**HR Per 1-SD Increment [95%CI]**	***p*-Value**
Crude	0.879 [0.639-1.210]	0.429
Corrected for gender	0.852 [0.604-1.201]	0.360
Corrected for age and gender	0.810 [0.583-1.125]	0.208
Corrected for age, gender, and ABI	0.821 [0.584-1.155]	0.257
Corrected for age, gender, and smoking	0.798 [0.573-1.112]	0.183

Data are expressed as hazard ratios (HR) per 1 standard deviation (SD) increment and their respective 95% confidence intervals (CIs).

Supplemental Table S5: Hazard ratios for mortality by triglycerides.

	HR Per 1-SD Increment [95%CI]	*p*-Value
Crude	0.976 [0.675-1.411]	0.897
Corrected for gender	0.989 [0.687-1.422]	0.951
Corrected for age and gender	1.185 [0.804-1.746]	0.392
Corrected for age, gender, and ABI	1.246 [0.843-1.842]	0.271
Corrected for age, gender, and smoking	1.118 [0.741-1.688]	0.595

Data are expressed as hazard ratios (HR) per 1 standard deviation (SD) increment and their respective 95% confidence intervals (CIs).

Supplemental Table S6: Hazard ratios for mortality by apolipoprotein A-I.

	HR Per 1-SD Increment [95%CI]	*p*-Value
Crude	0.754 [0.516-1.103]	0.146
Corrected for gender	0.768 [0.524-1.127]	0.178
Corrected for age and gender	0.754 [0.505-1.125]	0.167
Corrected for age, gender, and ABI	0.707 [0.463-1.081]	0.109
Corrected for age, gender, and smoking	0.769 [0.526-1.203]	0.279

Data are expressed as hazard ratios (HR) per 1 standard deviation (SD) increment and their respective 95% confidence intervals (CIs).

Supplemental Table S7: Hazard ratios for mortality by apolipoprotein B.

	HR Per 1-SD Increment [95%CI]	*p*-Value
Crude	0.830 [0.584-1.182]	0.302
Corrected for gender	0.843 [0.594-1.198]	0.342
Corrected for age and gender	0.758 [0.528-1.089]	0.134
Corrected for age, gender, and ABI	0.773 [0.534-1.121]	0.175
Corrected for age, gender, and smoking	0.769 [0.539-1.098]	0.149

Data are expressed as hazard ratios (HR) per 1 standard deviation (SD) increment and their respective 95% confidence intervals (CIs).

Supplemental Table S8: Hazard ratios for mortality by lipoprotein(a).

	HR Per 1-SD Increment [95%CI]	*p*-Value
Crude	1.166 [0.866-1.570]	0.312
Corrected for gender	1.144 [0.851-1.538]	0.374
Corrected for age and gender	1.042 [0.753-1.441]	0.805
Corrected for age, gender, and ABI	1.030 [0.739-1.437]	0.861
Corrected for age, gender, and smoking	1.028 [0.742-1.423]	0.869

Data are expressed as hazard ratios (HR) per 1 standard deviation (SD) increment and their respective 95% confidence intervals (CIs).

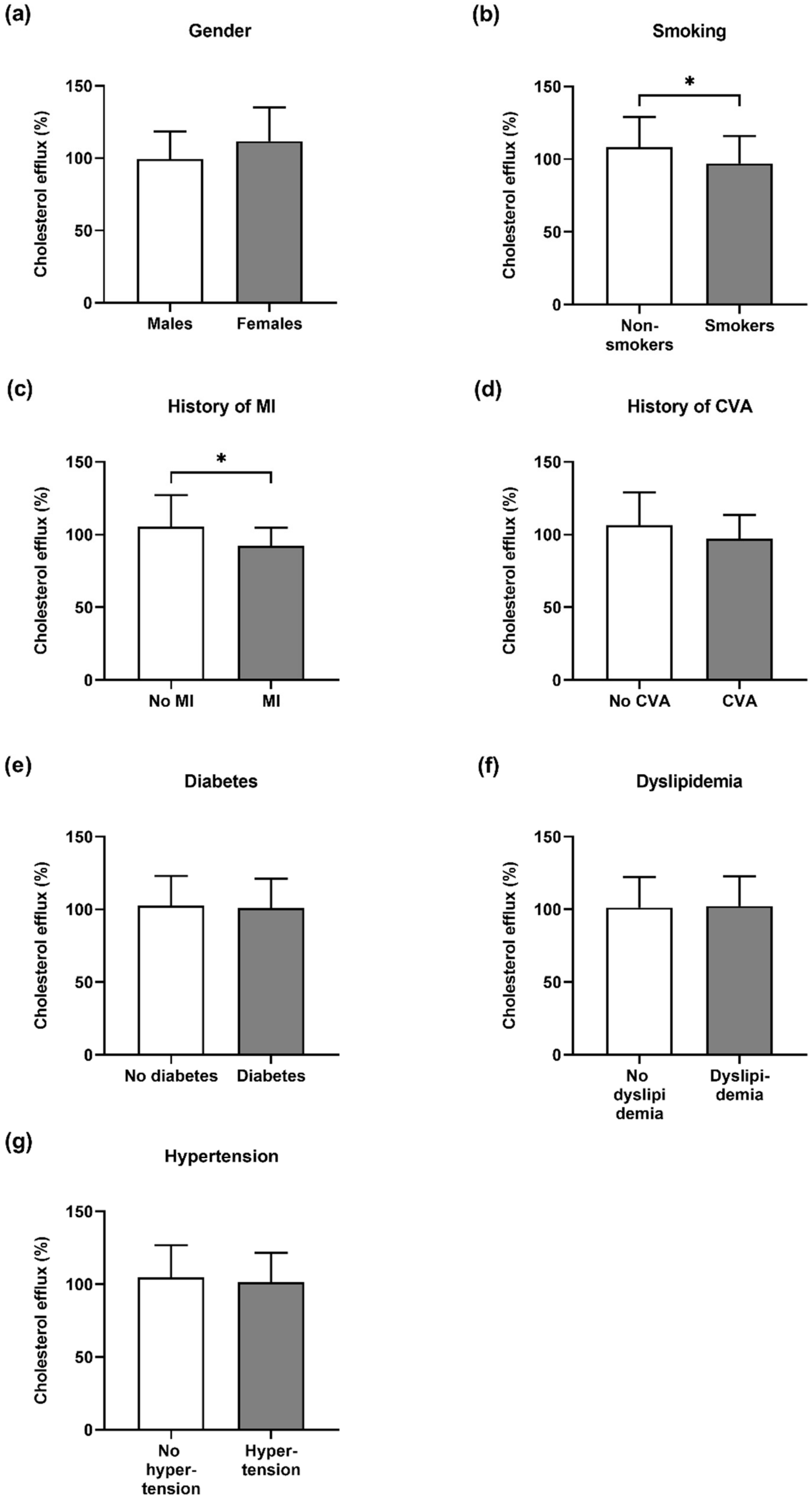

Supplemental Figure S1. CEC according to the clinical characteristics (a) gender, (b) smoking, (c) history of myocardial infarction (MI), (d) history of a cerebrovascular accident (CVA), (e) diabetes, (f) dyslipidemia, and (g) hypertension.
